# Early Evidence of Post-Mortem Fetal Extrusion in Equids: A Case from the Western Zhou Period (1045–771 BC) Site of Yaoheyuan in Northwestern China

**DOI:** 10.3390/ani14142106

**Published:** 2024-07-18

**Authors:** Zexian Huang, Qiang Ma, Chengrui Zhang, Ruoxin Cheng, Furen Hou, Yi Wu, Feng Luo, Yue Li

**Affiliations:** 1 Collaborative Research Centre for Archaeology of the Silk Roads, Northwest University, Xi’an 710127, China; huangzexian@stumail.nwu.edu.cn; 2 School of Cultural Heritage, Northwest University, Xi’an 710127, China; lf200862@126.com (F.L.); 3 Ningxia Institute of Cultural Relics and Archaeology, Yinchuan 750001, China; 4 Department of Anthropology, Harvard University, Cambridge, MA 02138, USA; 5 Beijing Institute of Archaeology (Beijing Institute of Cultural Heritage), Beijing 100009, China; wuyi_25@163.com

**Keywords:** China, Bronze Age, chariot-horse pit, horse, post-mortem fetal extrusion, zooarchaeology

## Abstract

**Simple Summary:**

The authors analyzed horse remains from a chariot-horse pit (CMK2) associated with elite burials at the Bronze Age site of Yaoheyuan in northwestern China. Among the horses interred in this specific pit, one adult female and one infant show evidence of post-mortem fetal extrusion. This conclusion is based on an examination of their age at death, sex, head orientation, and spatial relationships. The parturition stage of the foal suggests that the interment of the female horse likely occurred in late spring or early summer. The relatively high temperature may have generated gas in the body of the pregnant mare, eventually leading to the extrusion of the fetus. This represents the first reported case of post-mortem fetal extrusion in equids from archaeological contexts.

**Abstract:**

Post-mortem fetal extrusion, also known as “coffin birth”, refers to the phenomenon where a fetus is pushed out of a deceased female due to pressure from decomposing gas in the abdominal cavity. While post-mortem fetal extrusion has been documented in humans at several archaeological sites, there are few reports of it occurring in non-human animals. In this study, we present a case of post-mortem fetal extrusion in equids observed in a chariot-horse pit (CMK2) at the Western Zhou period site of Yaoheyuan in northwestern China, dating to the early first millennium BC. This specific pit, one of four excavated at the site, contained at least 29 horses and 3 wooden chariots. Most of these horses were young adults aged between 4 and 12 years. Out of the 22 horses with sex estimates, 21 were males. Among these individuals, one adult female horse (Horse 6) and one infantile horse (Horse 10) were of particular importance. Based on the age-at-death, sex, and head orientation of the two individuals, alongside their spatial relationships, it is highly likely that Horse 6 was the fetus of Horse 10 and was extruded in the pit. According to the parturition stage of Horse 10, Horse 6 was likely interred in CMK2 in late spring or early summer of the year, during which the relatively high temperature may have generated gas that led to the extrusion of the fetus. Although the specific reason for the inclusion of a pregnant mare in a chariot-horse pit at Yaoheyuan remains a topic for future research, this case marks the first report of post-mortem fetal extrusion in archaeological horses. The findings offer insights into the timing of horse interment as part of ritual practices among the settled elites during the Bronze Age in China and provide valuable reference data for contemporary equine veterinary science.

## 1. Introduction

Parturition marks the final stage of pregnancy in humans and non-human mammals, during which the newborn is delivered from the mother into the world [1]. This biological process is essential for the reproduction and continuation of mammal species. Normally happening while the mother is alive, parturition can, in very rare cases, occur after the mother’s death. This phenomenon, known as post-mortem fetal extrusion or “coffin birth”, happens when decomposing gases in the abdominal cavity exert pressure, leading to the extrusion of the fetus from the deceased mother’s body [2].

Despite their rarity, cases of post-mortem fetal extrusion have been documented in modern forensic science [3,4]. In the archaeological record, certain individual burials contain the skeletal remains of both females and fetuses. While some of these cases are attributed to complications during childbirth, such as preterm birth or obstructed labor, others are identified as post-mortem deliveries [5,6]. Examples from the latter category include, but are not limited to, a burial site of the early Neolithic period in Russia [6], Roman burials dating from the 3rd to 2nd centuries BC in Italy [7], medieval religious burials in Italy and Spain [8,9], mummified human remains of the 19th century in Finland [10], and a 19th-century public cemetery in Mauritius [11].

A common feature among these cases is that the fetuses are found in the pelvic and femoral positions of the mothers. However, unlike typical burials, the orientation of the fetuses’ heads is opposite to that of their mothers. This is a crucial characteristic in identifying post-mortem fetal extrusion. These findings yield important data for understanding ancient population demographics, maternal mortality rates, social customs, and mortuary practices [8,12,13].

While documented instances of post-mortem deliveries exist in humans from archaeological contexts, reports of similar phenomena in non-human animals are scarce. Several factors contribute to this rarity. Animals found in archaeological contexts can serve practical and ritualistic purposes. Bones from animals used for everyday consumption are usually unearthed from residential areas of sites and are often fragmented. Ritual uses may involve whole animals or specific body parts. The occurrence of post-mortem delivery depends fundamentally on the death of the pregnant mother, which is rare. In addition, the preservation of juvenile animals in archaeological contexts, particularly fetuses, is significantly influenced by environmental conditions. Their bones, which may be incompletely ossified and fragile, are prone to decay. Factors such as soil pH, temperature fluctuations, humidity, and microbial activity may further affect bone preservation. The excavation process also plays a crucial role, as excavator bias, excavation methods, and procedures can potentially lead to the misidentification or loss of fetal skeletal remains among fragmented bones.

Here, we present an analysis of the skeletal remains of an adult mare and a fetal horse found in a chariot-horse pit at the Bronze Age site of Yaoheyuan in northwestern China. This case represents the earliest documented evidence of post-mortem fetal extrusion in equids worldwide. These findings offer significant insights into understanding this phenomenon in non-human animals and shed light on the utilization, management strategies, and mortuary practices of horses in the northwestern frontier regions of the Western Zhou Dynasty during the early first millennium BC.

## 2. The Site of Yaoheyuan and the Chariot-Horse Pit CMK2

### 2.1. Yaoheyuan

In the mid-11th century BC, the Zhou people overthrew the Shang Dynasty and established the Western Zhou Dynasty [14,15]. To consolidate control over their newly acquired territories, the Zhou authority appointed members of the *Ji* clan and their allies to establish regional political entities. Through political alliances and military outposts, the Zhou authority exerted control over extensive areas [16,17]. In this context, the archaeological fieldwork at Yaoheyuan is of particular importance.

**Figure 1:**
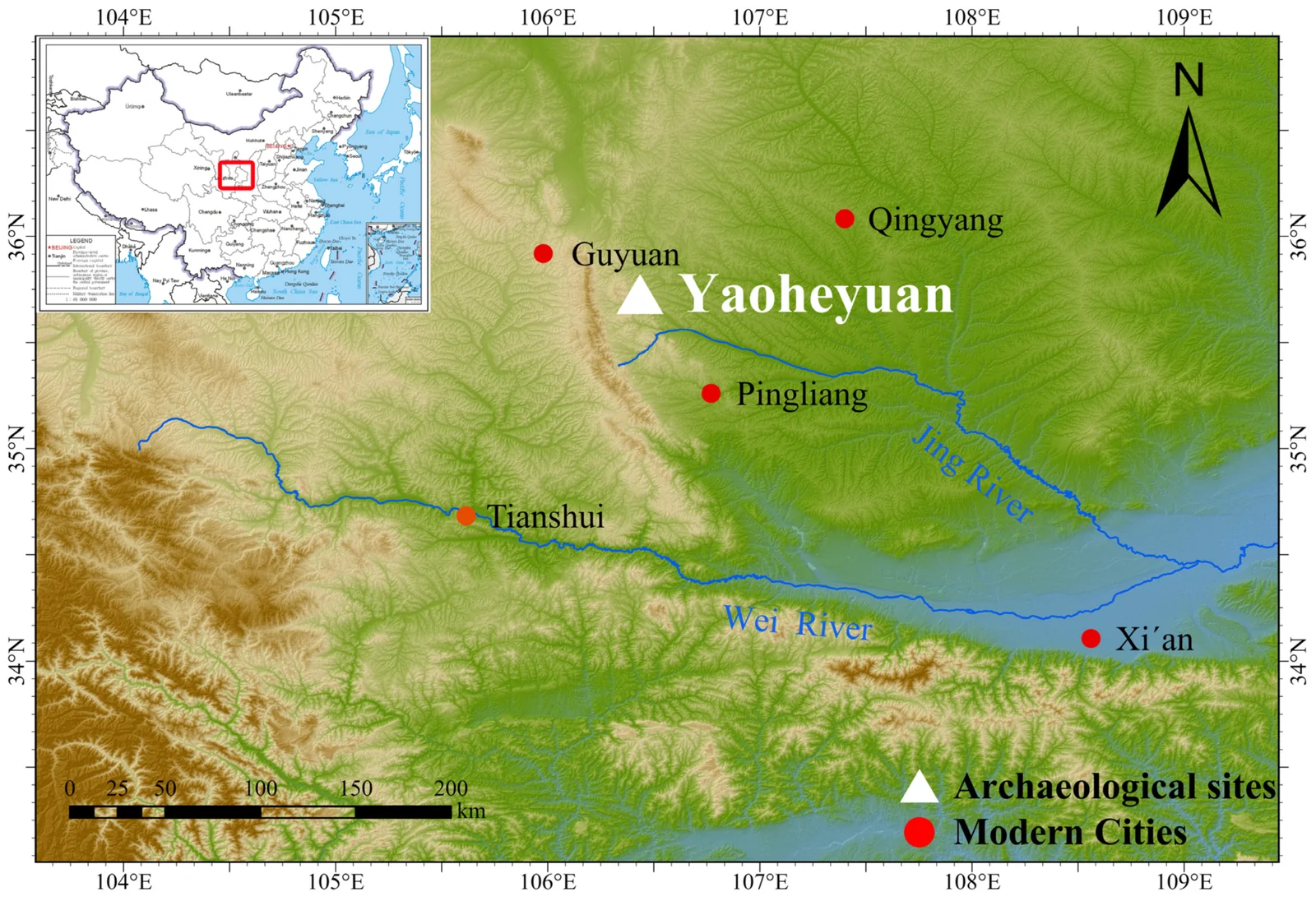
The location of the Yaoheyuan site in northwestern China. The digital elevation model (DEM) was acquired from the Geospatial Data Cloud site, Computer Network Information Center, Chinese Academy of Sciences (http://www.gscloud.cn, accessed on 27 May 2024). The map was produced in ArcMap 10.7.1.

The site of Yaoheyuan sits at the eastern foot of the Liupan Mountain (also known as the Longshan Mountain), in the upper reaches of the Jing River, in the present-day Ningxia Hui Autonomous Region (see Figure 1). The location of Yaoheyuan is adjacent to the Guanzhong region in modern central Shaanxi Province, which served as the royal domain of the Western Zhou Dynasty [18].

**Figure 2:**
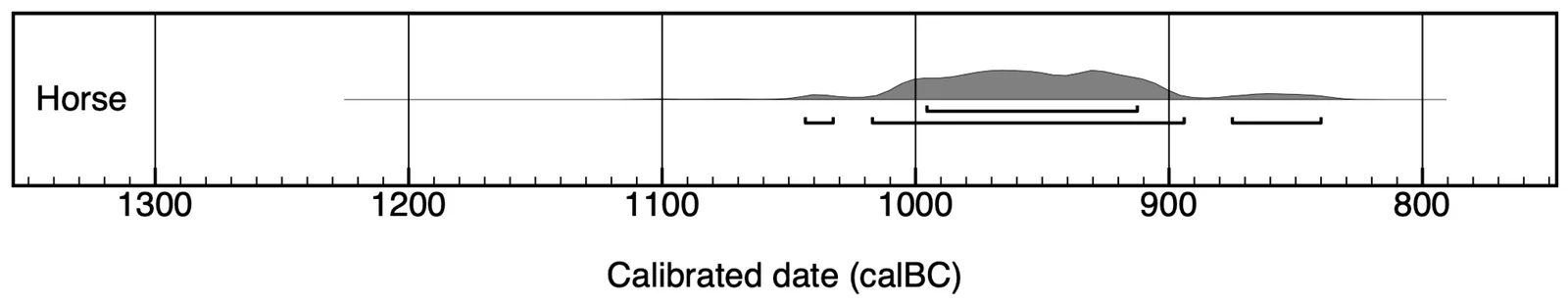
The radiocarbon dating result for Horse 14. Conventional radiocarbon age (2800 ± 30 BP, Beta-647876) was generated by the Beta Analytic Radiocarbon Dating Laboratory. Calibration was performed in OxCal v.4.4 [21], using the IntCal20 calibration curve [22].

Covering an area of approximately 96 ha, Yaoheyuan is characterized by a walled inner city (56 ha) and an adjacent outer city (40 ha) [18]. Archaeological surveys and excavations in previous years have revealed various features, such as a moat, walls, burials, a palace foundation, workshops, and roads [18,19,20]. While high-ranking burials, the palace foundation, the bronze casting workshop, and ceramic production workshops were located within the inner city, the outer city contained primarily trash pits, storage pits, houses, kilns, and roads. The artifacts unearthed include ceramics, bronze vessels, proto-celadon vessels, jade and lithic artifacts, inscribed oracle bones, as well as bone, antler, and ivory products [18]. Based on oracle bone inscriptions, Yaoheyuan may have been the capital of the *Huo* state, a vassal state established in the early Western Zhou period [20]. This is also consistent with the radiocarbon dating result from one fragment of horse remains, which places the context in the early Western Zhou period (Figure 2).

Located in the northwestern frontier of the Western Zhou Dynasty, Yaoheyuan likely served as a military outpost of the Zhou authority [20]. The site is one of the most significant new discoveries of the Western Zhou period. The site represents the westernmost early Zhou vassal state capital. It contains the westernmost early Zhou high-ranking elite burials and bronze casting workshop and yields the westernmost oracle bones and proto-celadon vessels. These findings provide new insights into the political landscape during the Western Zhou period and the relationships between the Zhou Dynasty and its northwestern frontiers [19].

### 2.2. The Chariot-Horse Pit

It appears that the residents at Yaoheyuan had access to a network for acquiring luxury items such as proto-celadon vessels, turquoise, and jade artifacts, and. Horses likely played an important role in these trade networks [18]. As key material resources, horses were crucial to the elites of the Western Zhou period for military and ritual occasions [23]. A large number of horse pits and chariot-horse pits, dated to the Western Zhou period, have been discovered across northern China over the past decades [24,25,26].

**Figure 3:**
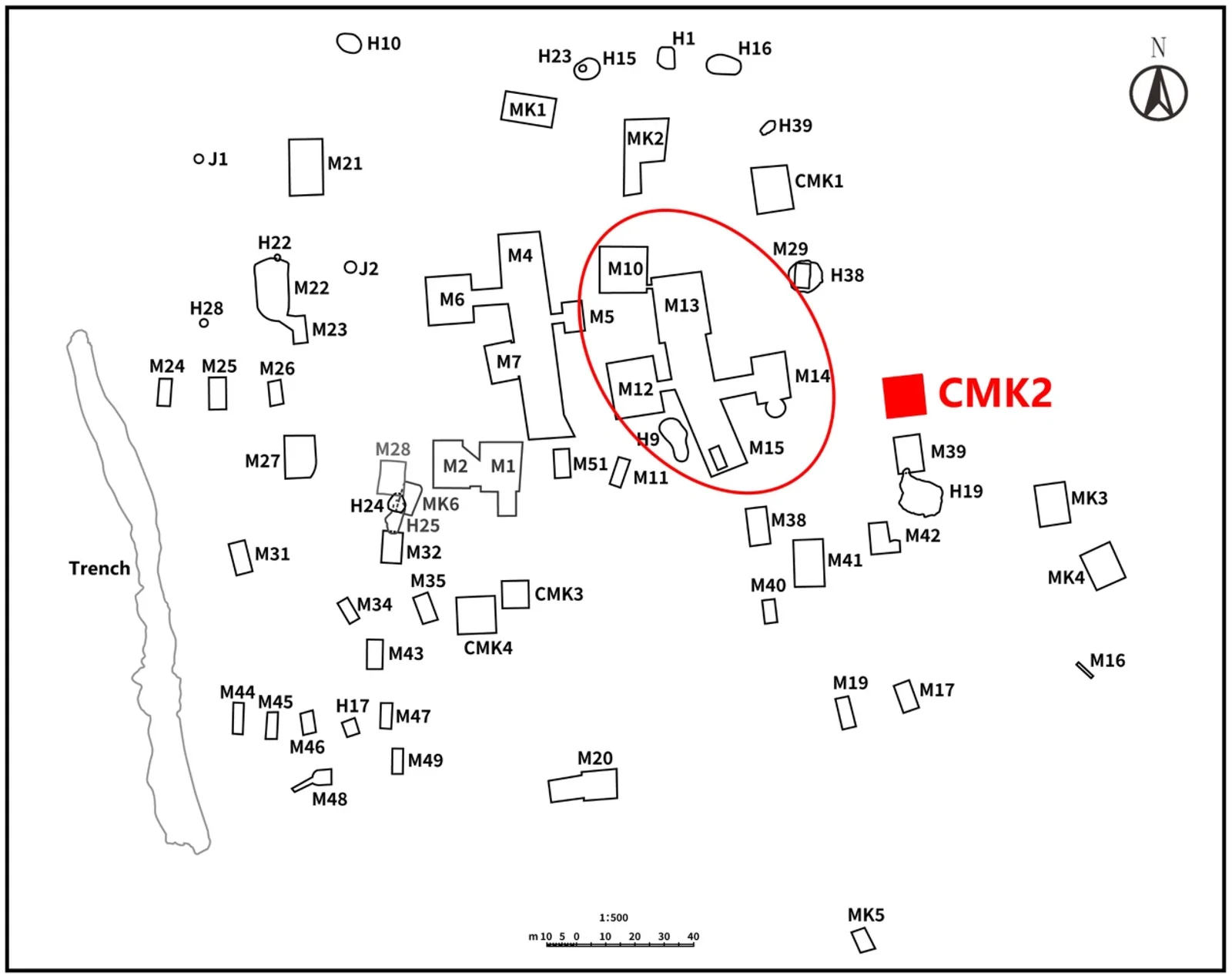
The location of CMK2 in Yaoheyuan. “M” refers to burial, “H” refers to pit, “J” refers to well, “MK” refers to horse pits, and “CMK” refers to chariot-horse pit.

As of 2021, six horse pits (designated as MK) and four chariot-horse pits (designated as CMK) have been excavated within the high-ranking burial area of Yaoheyuan [18]. Located on the eastern edge of the high-ranking burial area, chariot-horse pit CMK2 is adjacent to the group burials M10, M12, M13, M14, and M15 (Figure 3). CMK2 has a nearly square opening, measuring 4 m in length from north to south, 4.2 m in length from east to west, and 3.1 m in depth. Inside CMK2, the chariots and horses are separated, with disassembled chariot elements placed above the horses. In the western and southeastern parts of CMK2, a number of chariot and horse fittings were unearthed. The absence of infilling between the chariots and the horses indicates that the horses may have been directly buried after the interment of the chariots. CMK2 contains the largest number of chariots and horses among all pits of this kind discovered at Yaoheyuan.

## 3. Materials and Methods

### 3.1. On-Site Extraction

Once all horse remains in CMK2 were exposed, we began the on-site extraction process. We documented the distribution, orientation, and quantity of the horses. Based on the overlapping relationships of the horses and the physiological characteristics of their skeletal elements, we identified the individuals buried in the pit later or relatively later. We then numbered the horses sequentially from the southeastern to the northwestern parts of the pit.

We recorded all of the information in as much detail as possible, including the position, posture, and relationships with surrounding individuals of the horses, as well as the preservation conditions of the bones. These horse remains were labeled using professional skeletal terminology to facilitate analysis of bone positions, particularly their relationships. Measurements of important skeletal parts were taken on-site. Detailed textual and visual records were made both before and after extraction.

### 3.2. The Zooarchaeological Analysis

Identification, recording, measurement, and quantitative statistical analyses were conducted on all available skeletal elements to gather data on sex, age at death, abnormalities, etc. The sex of the horses was determined primarily based on the presence/absence and characteristics of the canines [27]. The age at death was assessed by examining the eruption and wear of the mandibular teeth [27]. Withers height, an important criterion for evaluating the quality of a horse refers to shoulder height—the vertical distance between the top of the scapula and the ground [28]. The estimation of withers height followed the methods proposed by Hayashida & Yamauchi [29] and May [30]. The slenderness index was calculatedto evaluate the robustness of horse limb bones [31].

## 4. Results

### 4.1. Horse Remains in CMK2

**Figure 4:**
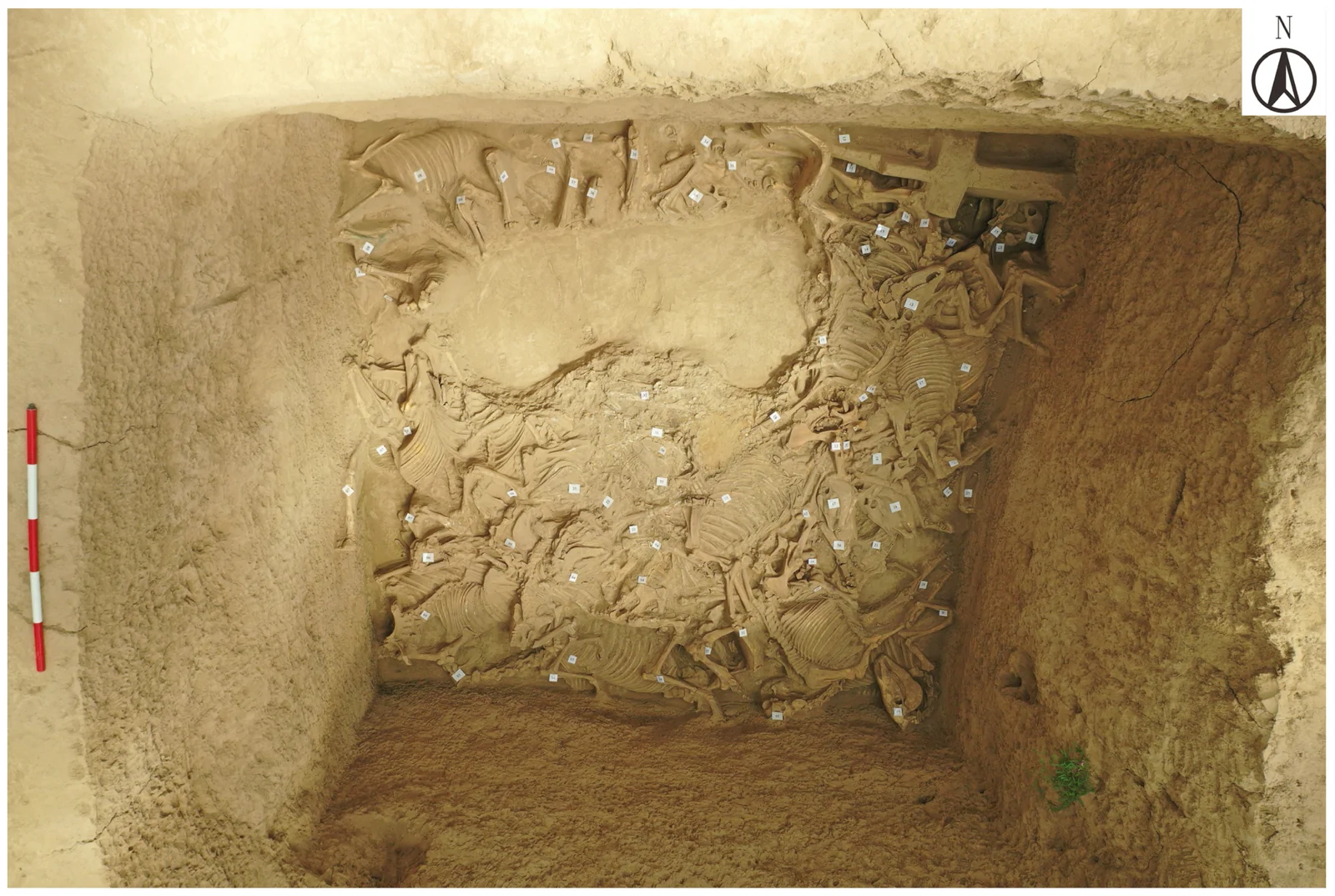
The horse remains in the chariot-horse pit CMK2 at Yaoheyuan.

All horses were found at the bottom of CMK2, with the northern part of the pit showing signs of looting, resulting in scattered and fragmented horse bones in the vicinity (Figure 4). The total count of horse bones, including those affected by looting, amounted to 3070 pieces, most of which were intact. At least 29 individuals were identified and numbered from “Horse 1” to “Horse 29”.

The horses in CMK2 were densely packed, with complex relationships and overlapping between individuals. There was no discernable pattern to their positioning; the heads of these horses were oriented in various directions. Most horses were found lying on their sides, while some were in a prone position. There were no indications of struggle, suggesting that the horses were likely buried post-mortem.

The interment sequence of all horses in CMK2 was reconstructed. These horses were roughly stratified into four layers, from bottom to top. In the fourth layer, there were 16 horses, most of them positioned close to the bottom of the pit. These horses were distributed in the eastern, southern, and western parts of the pit’s bottom, indicating they were the earliest individuals buried. The third layer contained 6 horses, stacked on top of those in the fourth layer, concentrated in the northeast part of the pit. The second layer contained a total of 5 individuals, primarily located in the eastern and southern parts of the pit. The first layer, at the topmost level, consisted of 2 horses found only in the northeast corner of the pit, representing the latest interments in the burial sequence.

The ages of 27 horse individuals in CMK2 were estimated. Among these, 11 individuals were younger than 4 years old, constituting 40.7% of all horses in CMK2. A total of 13 horses were between 4 and 12 years of age, making up nearly 50.0%. The remaining 3 horses were older than 12 years, accounting for 11.1% of the total. It appears that the majority of horses in CMK2 were young adults aged between 4 and 12 years, followed by those under 4 years old, with relatively fewer individuals over 12 years old. Out of the 22 horses with sex estimates, all except one were males [32].

### 4.2. Horse 6 and Horse 10

**Figure 5:**
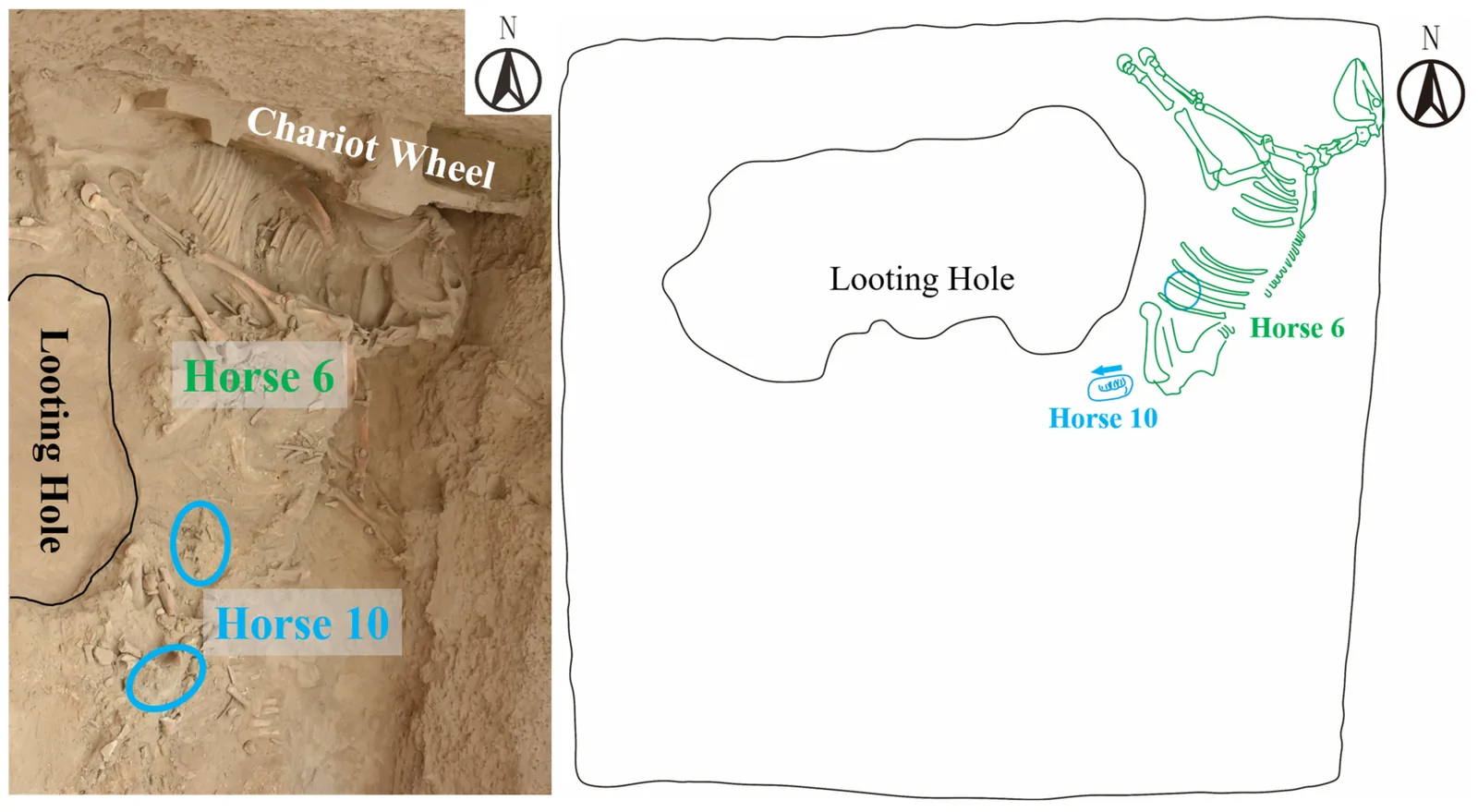
The location of Horse 6 and Horse 10 in CMK2. The blue circle in the left half of the figure marks the location of the remains of Horse 10. The arrow in the right half of the figure shows the orientation of Horse 10’s head.

We identified one adult female horse (ID: Horse 6) at the northeastern corner of CMK2. Adjacent to the pelvis and femora of this individual, slightly to the southwest, there was one extremely young individual (ID: Horse 10) (Figure 5).

**Figure 6:**
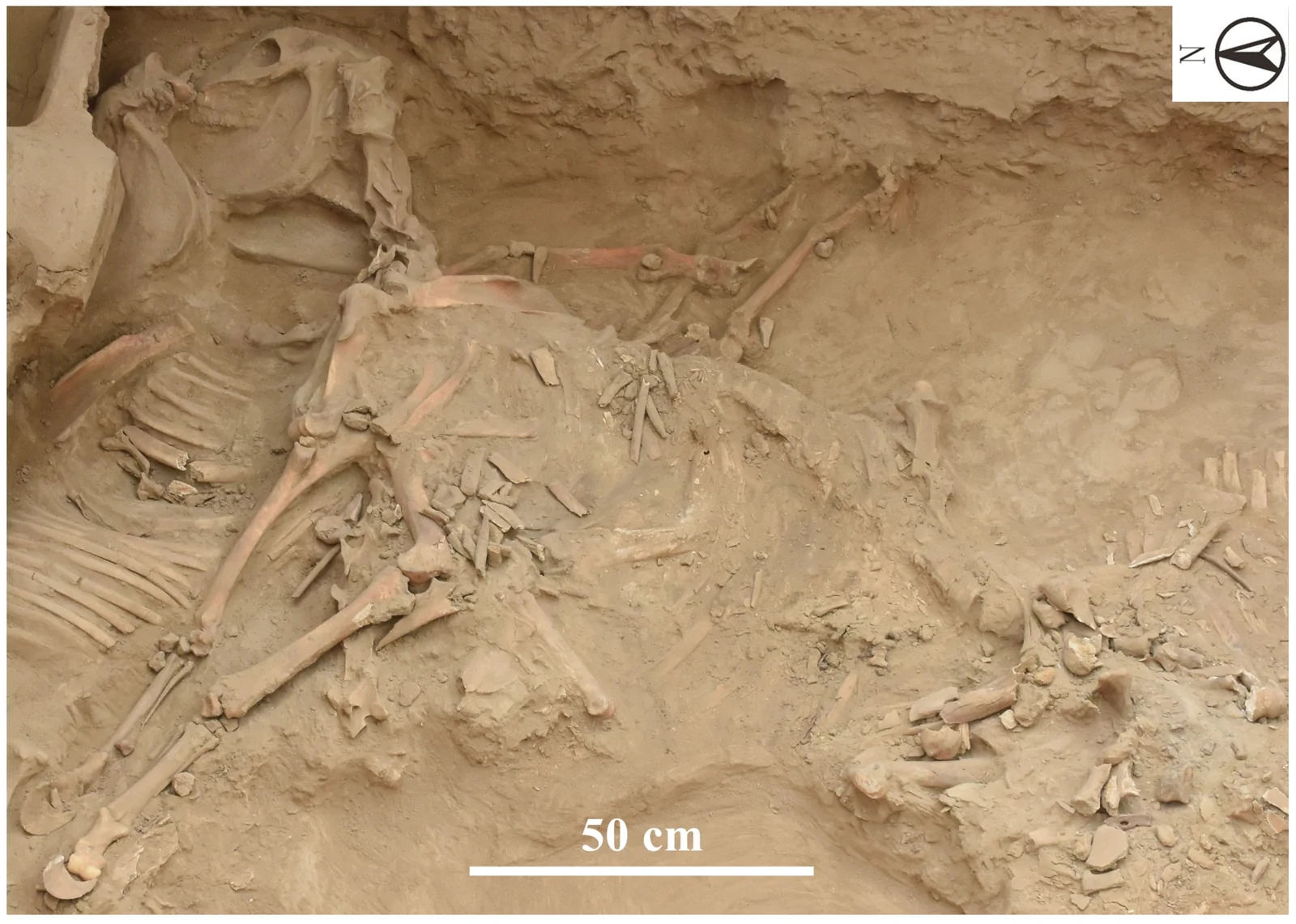
The remains of Horse 6 in CMK2.

More specifically, Horse 6 is positioned at the east edge of the looting hole. The head of Horse 6 faces north, while its tail points southwest. The horse is lying on its side, with the left side facing upwards. The occipital bone is against the eastern wall of CMK2, approximately 67 cm away from the northern wall. Horse 6 is nearly intact, with the skull and forelimbs relatively well-preserved. However, the vertebrae and ribs are fragmented, and only the pelvis and femora remain, as the tibia and tarsal bones have been disturbed by looting (Figure 6).

Based on the eruption and wear of the mandibular teeth, Horse 6 was determined to be 15 years old. The absence of canines suggests that this was a female horse. Two calculation methods [29,30] were applied to the humerus, radius, and metacarpal bone of Horse 6 to estimate the withers height. The mean withers height was 137.7 cm (Table 1).

The metacarpal bone’s minimum width (SD) and greatest length (GL) were used to calculate the slenderness index, using the formula SD/GL×100 [33,34]. The slenderness is classified into six categories: very slender-legged, slender-legged, slightly slender-legged, medium slender-legged, slightly massive-legged, and massive-legged [33,34]. After calculation, the score of the metacarpal slenderness index for Horse 6 is 14.07, which falls into the “slender-legged” category.

**Figure 7:**
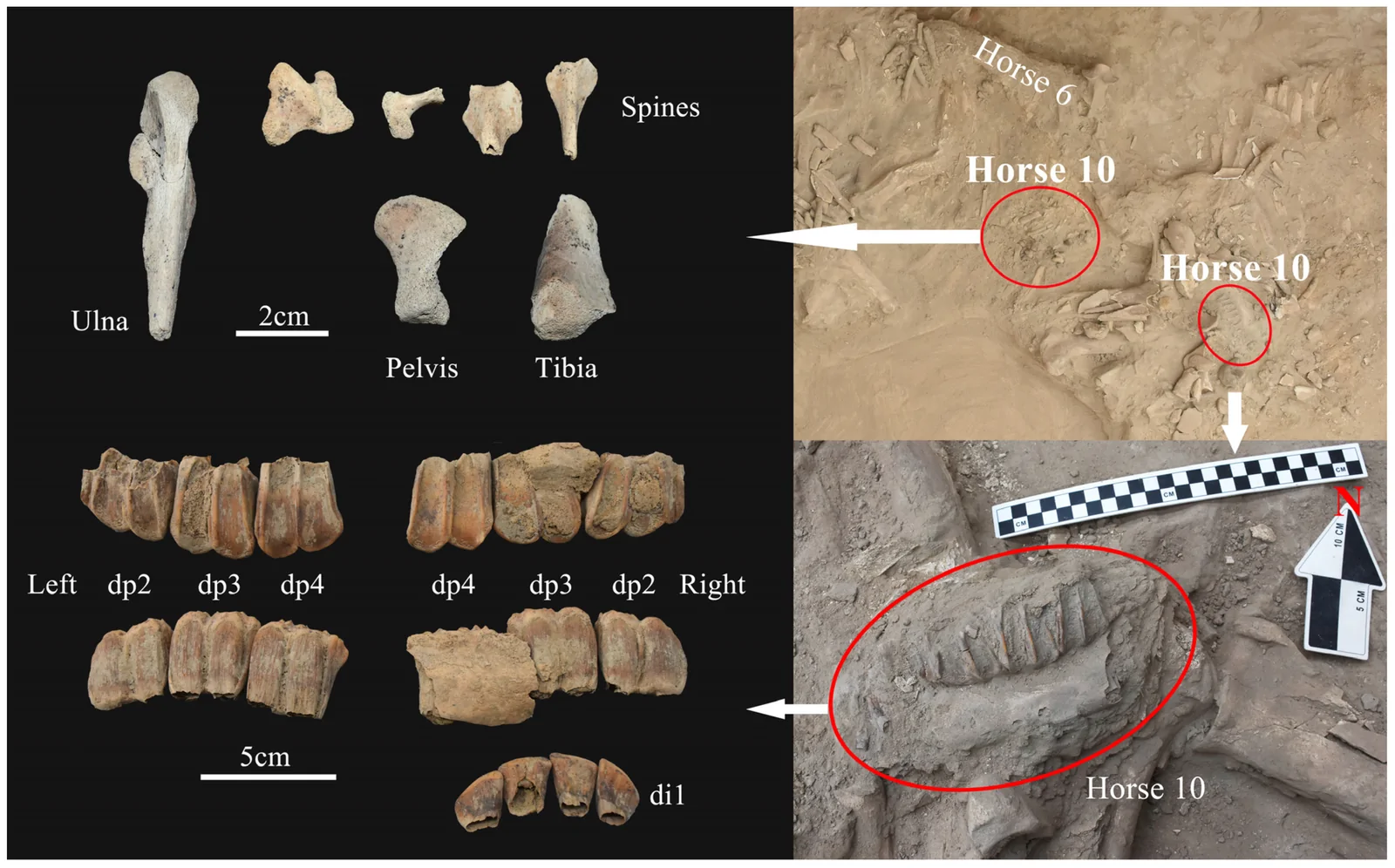
The remains of Horse 10 in CMK2. The right half of Figure 7 shows the on-site excavation of Horse 10, with its skeletal remains indicated within the red circle. Vertebrae and limb bone fragments of Horse 10 are found in the abdomen of Horse 6, and the skull of Horse 10 lies behind the pelvis of Horse 6. Close-ups of the teeth and other skeletal elements of Horse 10 are shown on the left half of Figure 7.

Horse 10, located in the eastern part of CMK2, is positioned at the southwest edge of the looting horse. Only one piece of each of the crania and mandibles, right radius, pelvic bone, and tibia were preserved, along with four fragments of vertebrae. The cranium and mandible are approximately 209 cm from the northern wall and 117 cm from the eastern wall of the pit, which are adjacent to the pelvic bone and femur of Horse 6, with the head facing southeast. Similar to Horse 6, Horse 10 is also lying on its side, with the left side facing upwards. A total of twelve teeth are visible, including four deciduous incisors (di1) on each side of the crania and mandibles and deciduous premolars dp2, dp3, and dp4. The limb bones and vertebrae fragments are located behind the abdomen of Horse 6. The thin and fragile bone walls indicate incomplete bone development. The remaining length of the radius fragment is only 82.03 mm, and the other fragments range from 2 to 4 mm in length, clearly indicating a young individual (Figure 7). 

According to modern equine dental morphology and radiographic studies [35], the morphology of Horse 10’s teeth closely resembles that of a horse fetus at 275 days of gestation. Therefore, the gestation period of Horse 10 is estimated to be no less than 9 months, indicating that Horse 10 is a fetus aged 9 to 11 months. Due to its very young age, the sex cannot be determined.

### 4.3. Ancient DNA Analysis

To further verify the genetic relationship between Horse 6 and Horse 10, we conducted an ancient DNA analysis of the petrosal bone of Horse 6, along with the skeletal fragments and teeth of Horse 10. Unfortunately, despite multiple extraction attempts, both double-stranded and single-stranded library constructions were unsuccessful due to the poor preservation of Horse 10.

## 5. Discussion

### 5.1. Post-Mortem Fetal Extrusion

According to modern livestock records, horses are monozygotic animals with a gestation period ranging from 330 to 340 days and a breeding age range of 3 to 15 years [36]. Horse 6 is the sole adult female individual in CMK2 whose sex can be determined. At the age of 15 years old, Horse 6 was at the upper limit of reproductive age. Meanwhile, Horse 10 is a fetus, with its skull positioned very close to the pelvic bone of Horse 6, indicative of imminent birth. Considering the age at death, sex, and positioning of these two individuals, it is highly likely that Horse 10 was delivered by Horse 6.

Given the rarity of reported cases from archaeological contexts where adult female horses give birth to very young individuals, understanding the specific reasons for the presence of Horse 6 and Horse 10 in CMK2 at Yaoheyuan requires referencing forensic and anthropological data. Archaeologists employ specific criteria to discern the phenomenon of maternal and fetal co-burials [13]. If the fetus’s bones are entirely within the mother’s pelvic cavity, it suggests fetal death before delivery, possibly due to complications such as hemorrhage or dystocia, which could have led to the mother’s death. Conversely, if the fetal skull faces the same direction as the mother’s, or if the legs are extended, or if the fetal skull rests near the mother’s ribs, it may indicate post-delivery death and deliberate placement. When fetal remains are relatively intact and outside the pelvis, with the skull of the fetus being oriented opposite to its mother’s, and after ruling out post-depositional effects, the possibility of post-mortem fetal extrusion might explain. It is important to note that sufficient space must be available for post-mortem delivery, as a fetus is unlikely to be expelled from the mother’s body in crowded conditions.

The burial of a female associated with a partially expelled fetus, discovered at archaeological sites, has been interpreted as the result of complications during parturition, including hemorrhage, prematurity, and primarily, dystocia [37]. However, as mentioned earlier, several studies suggest that post-mortem fetal extrusion can also lead to the fetus being positioned near the mother’s pelvic bones. Forensic evidence also indicates that as organic tissues of a corpse begin to decompose and decay, gases are produced, causing abdominal bloating [38]. This bloating increases intra-abdominal pressure, which leads to the relaxation of the soft tissues and the potential expelling of the fetus from the mother’s body, particularly in the pelvic region [39].

**Figure 8:**
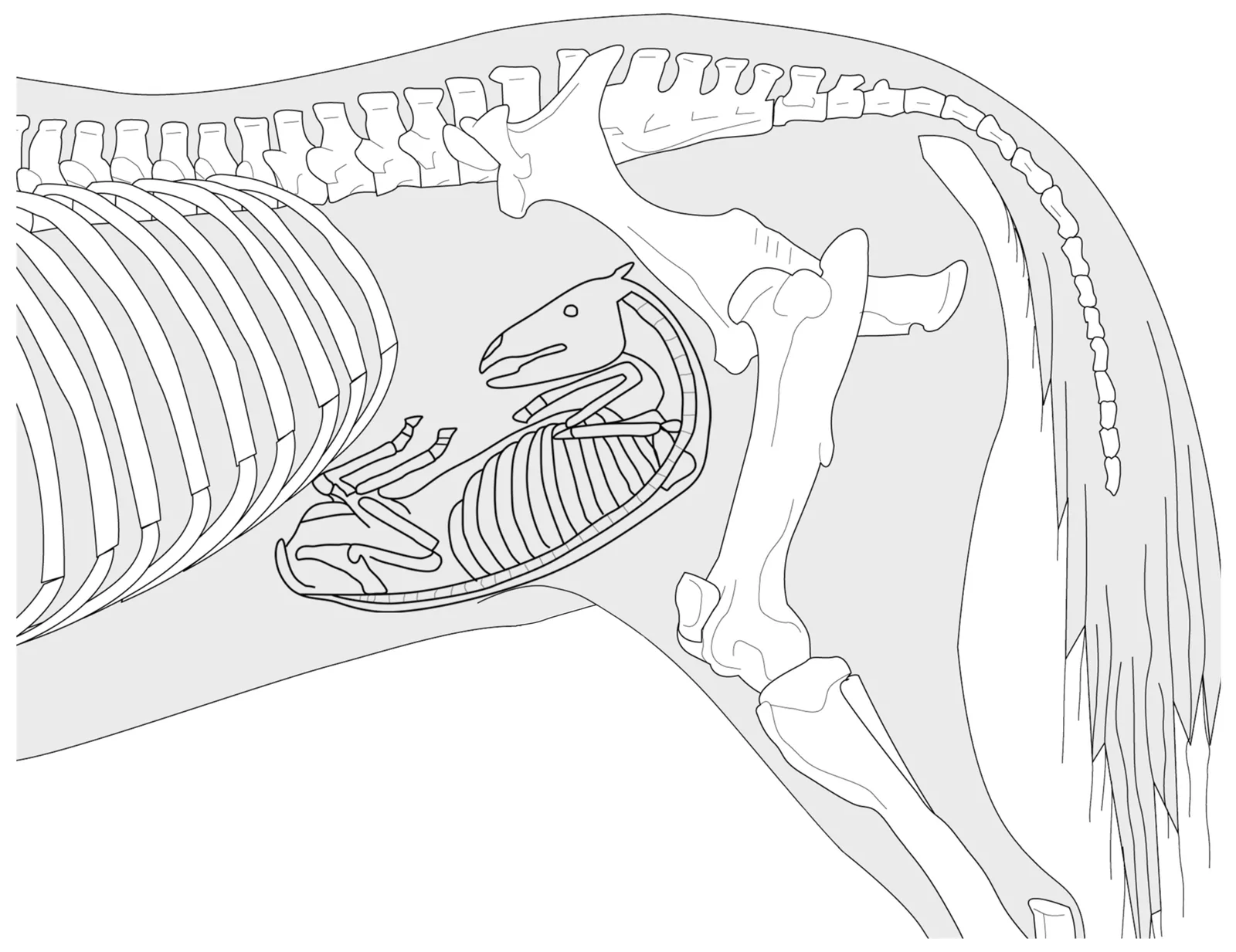
The position of a fetus in a pony mare at about approximately 300 days gestation (modified from Figure 4 in [38]).

**Figure 9:**
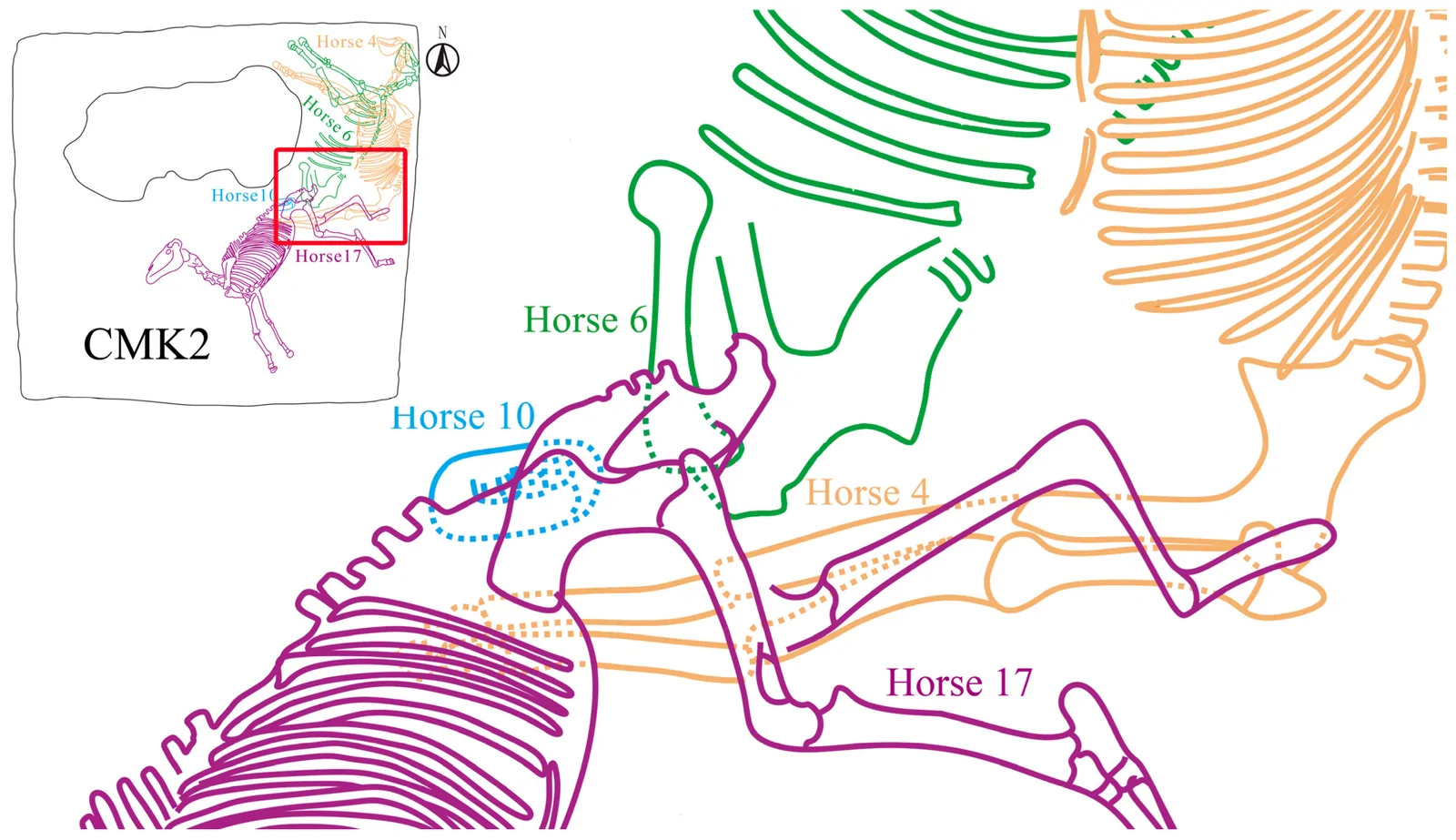
The spatial relationship between Horse 6 and Horse 10. The front half of Horse 4 is positioned against the forelimbs of Horse 6, while its hindlimbs are placed behind Horse 6’s pelvis without pressing down on it. Meanwhile, the hindlimbs of Horse 17 are pressed against the hindlimbs of Horse 4. Before flesh decayed, it is likely that the buttocks and hindlimbs of Horse 17 were supported by the hindlimbs of Horse 4, suspending them above Horse 6. This arrangement created sufficient space around the birth canal of Horse 6, which remained uncovered by soil, facilitating the delivery of Horse 10.

The skull of Horse 10 is positioned southwest of the caudal part of Horse 6’s pelvis and the proximal end of its femur, while vertebral fragments and limb bones are found in Horse 6’s abdomen. If Horse 10 had died due to dystocia or other complications, its skull and limb bones would likely be concentrated near Horse 6’s pelvis. However, the distance between the skull and limb bones of Horse 10 exceeds the distance of anatomical positions. Veterinary studies indicate that during late pregnancy, most horses assume a supine position with their heads directed toward the birth canal [40] (Figure 8). Horse 10 faces southwest, opposite to Horse 6, aligning with the direction of the birth canal of Horse 6. Considering their positions and head orientations, this arrangement corresponds with the characteristics of post-mortem fetal extrusion. In terms of space, directly above Horse 10 are the metatarsal bones of Horse 4 (male) and the pelvis and sacral vertebrae of Horse 17 (male). This spatial arrangement ensures that the infilling would not obstruct the birth canal of Horse 6, allowing sufficient space for the fetus to be expelled from the mother’s body (Figure 9).

Taken together, the age-at-death, sex, head orientation, and stage of delivery of Horse 6 and Horse 10, alongside their spatial relationship suggest that Horse 10 was a fetus delivered post-mortem by Horse 6.

### 5.2. The Season of Horse Interment

Horses are seasonal breeding animals. In the Northern Hemisphere, the estrus period of horses typically occurs in the spring and summer months (March–August), during which they are able to conceive. After an average gestation period of approximately 330 days (around 11 months), horses give birth [41]. Pregnancy, fetal development, and birth are influenced by genetic, maternal, and environmental factors. Seasonal variations in the mare’s metabolism also impact fetal growth. Modern veterinary studies have shown that foals born in early spring consistently have lower heights compared to those born in late spring [42]. Foals conceived between February and May and born the following spring benefit from lush pastures and the robust lactation abilities of the mares, resulting in better development. However, the hot weather from June to August can disturb mare reproductive activities, leading to lower conception rates. In present-day China, the period of April to June is considered the peak breeding season for mares to enhance conception rates and produce healthier foals [43].

During the Western Zhou period, horses served not only as crucial military assets but also as integral components of social and economic systems that encompass rituals and trade. Inscriptions on Western Zhou bronze vessels frequently detail the appearances and breeding locations of horses [44]. The *Zhouli* (*Rites of Zhou*) documents the establishment of official positions such as *Wuma* and *Mazhi*, tasked with the feeding and management of horses for the Zhou Dynasty [45]. Alongside the discovery of numerous horse pits and chariot-horse pits from the Western Zhou period across northern China [24,25,26,46], these lines of evidence underscore the significance and organized nature of horse-related activities during the Zhou era.

According to the *Liji* (*Book of Rites*), in the third month of spring, which corresponds to April-May in the Gregorian calendar, cattle and horses were allowed to mate freely on the pastures, and the number of quality foals and calves was recorded. In the second month of summer, which corresponds to June-July in the Gregorian calendar, pregnant mares were separated from stallions for grazing, and young colts capable of leaping were restrained. Orders pertaining to horse breeding were issued [47]. Although the compilation of the *Liji* was relatively late and may not precisely reflect horse breeding practices during the Western Zhou period, these records suggest that the Zhou people managed horse breeding and reproduction in accordance with seasonal climate changes.

Taking historical textual records into account, mare Horse 6 in CMK2 at Yaoheyuan may have mated and become pregnant in late spring to early summer (April–June). The presence of Horse 10, a fetus with a gestation period of approximately 9–11 months, suggests that both horses likely died between January and May of the following year. In temperate climates and enclosed spaces, decomposition of body organs typically occurs within 3–6 days in summer and within 3–6 weeks in winter [48]. Moreover, some researchers argue that post-mortem delivery must occur within 48–72 h of death, as prolonged decomposition of fetal remains would prevent extrusion [8]. Therefore, it is plausible that Horse 6 and Horse 10 died during the relatively warmer months (April to May), falling within the spring to the summer period.

Based on the accumulation and thickness of buried horses, it appears that the interment of horses in CMK2 was a one-time event, with no evidence of multiple burials. The absence of signs of struggle or limb binding in the horses’ postures and positions suggests that they were likely killed elsewhere and buried directly in the chariot-horse pit. In addition, given the positions of some horses and the apparent bending of many skeletal remains, it is likely that these horses died and were buried in close succession. Combining this with the timing of death for Horse 6 and Horse 10, it is probable that the horses in CMK2 were interred between late spring and early summer.

During the Western Zhou period, chariot-horse pits were exclusive to elite burials. At Yaoheyuan, CMK2 is likely one of the pits associated with nearby group burials (M10, M12, M13, M14, and M15) centered around M13. This suggests that one of the deceased individuals in the group burials may also have been interred between late spring and early summer.

## 6. Conclusions

The age at death, sex, burial positions, and time of death of two horse individuals (Horse 6 and Horse 10) in chariot-horse pit CMK2 at the Western Zhou (1045–771 BC) period site of Yaoheyuan in northwestern China have been documented. Horse 10 was a fetus in utero, resulting from a post-mortem fetal extrusion of Horse 6 between late spring and the early summer of the year. This discovery marks the first reported case of post-mortem delivery in non-human animals at archaeological sites worldwide.

The identification of post-mortem fetal extrusion in horses at Yaoheyuan owes much to the involvement of zoo archaeologists in the excavation and extraction process of horse remains, as well as the meticulous documentation of relevant data on-site. This unique finding provides a valuable case study for understanding post-mortem delivery in non-human animals, as well as the utilization, management strategies, and mortuary practices of horses in Bronze Age China. It offers important insights for future excavations and future zooarchaeological and veterinary research.

## Figures and Tables

**Table 1:** Estimates of the withers height for Horse 6 (unit: cm).

	Humerus	Radius	Metacarpal	Mean Value
GL (Greatest Lenth)	28.9	34.4	22.5	
Hayashida and Yamauchi’s method	135.1	140.9	137.4	137.8
May’s method	133.9	141.4	137.3	137.5
**Mean withers height**				**137.7**

## Data Availability

All relevant data are included in this manuscript.
