# Correction: Huang et al. Early Evidence of Post-Mortem Fetal Extrusion in Equids: A Case from the Western Zhou Period (1045–771 BC) Site of Yaoheyuan in Northwestern China. *Animals* 2024, *14*, 2106

**DOI:** 10.3390/ani16182940

**Published:** 2026-09-18

**Authors:** Zexian Huang, Qiang Ma, Chengrui Zhang, Ruoxin Cheng, Furen Hou, Yi Wu, Feng Luo, Yue Li

**Affiliations:** 1 Collaborative Research Centre for Archaeology of the Silk Roads, Northwest University, Xi’an 710127, China; huangzexian@stumail.nwu.edu.cn; 2 School of Cultural Heritage, Northwest University, Xi’an 710127, China; 3 Ningxia Institute of Cultural Relics and Archaeology, Yinchuan 750001, China; 4 Department of Anthropology, Harvard University, Cambridge, MA 02138, USA; 5 Beijing Institute of Archaeology (Beijing Institute of Cultural Heritage), Beijing 100009, China

Error in Citations

In the original publication [1], the citation numbers were erroneously shifted forward by one, leading to incorrect references.

The citations have now been corrected in 5. Discussion, 5.2. The Season of Horse Interment, Paragraphs 1–2, and should read as follows:

Horses are seasonal breeding animals. In the Northern Hemisphere, the estrus period of horses typically occurs in the spring and summer months (March–August), during which they are able to conceive. After an average gestation period of approximately 330 days (around 11 months), horses give birth [41]. Pregnancy, fetal development, and birth are influenced by genetic, maternal, and environmental factors. Seasonal variations in the mare’s metabolism also impact fetal growth. Modern veterinary studies have shown that foals born in early spring consistently have lower heights compared to those born in late spring [42]. Foals conceived between February and May and born the following spring benefit from lush pastures and the robust lactation abilities of the mares, resulting in better development. However, the hot weather from June to August can disturb mare reproductive activities, leading to lower conception rates. In present-day China, the period of April to June is considered the peak breeding season for mares to enhance conception rates and produce healthier foals [43].

During the Western Zhou period, horses served not only as crucial military assets but also as integral components of social and economic systems that encompass rituals and trade. Inscriptions on Western Zhou bronze vessels frequently detail the appearances and breeding locations of horses [44]. The *Zhouli* (*Rites of Zhou*) documents the establishment of official positions such as *Wuma* and *Mazhi*, tasked with the feeding and management of horses for the Zhou Dynasty [45]. Alongside the discovery of numerous horse pits and chariot-horse pits from the Western Zhou period across northern China [24–26,46], these lines of evidence underscore the significance and organized nature of horse-related activities during the Zhou era.

Text Correction

There was an error in the original publication [1]. Due to incorrect citation marking, some text requires revision.

A correction has been made to 1. Introduction, Paragraph 2, and should read as follows:

In the archaeological record, certain individual burials contain the skeletal remains of both females and fetuses. While some of these cases are attributed to complications during childbirth, such as preterm birth or obstructed labor, others are identified as post-mortem deliveries [5,6]. Examples from the latter category include, but are not limited to, a burial site of the early Neolithic period in Russia [6], Roman burials dating from the 3rd to 2nd centuries BC in Italy [7], medieval religious burials in Italy and Spain [8,9], mummified human remains of the 19th century in Finland [10], and a 19th-century public cemetery in Mauritius [11].

Additionally, a correction has been made to 5. Discussion, 5.2. The Season of Horse Interment, Paragraph 4, and should read as follows:

Moreover, some researchers argue that post-mortem delivery must occur within 48–72 h of death, as prolonged decomposition of fetal remains would prevent extrusion [8].

The authors state that the scientific conclusions are unaffected. This correction was approved by the Academic Editor. The original publication has also been updated.
